# Exploring Genomic Regions Associated with Fruit Traits in Pepper: Insights from Multiple GWAS Models

**DOI:** 10.3390/ijms252111836

**Published:** 2024-11-04

**Authors:** Nayoung Ro, Hyeonseok Oh, Ho-Cheol Ko, Jungyoon Yi, Young-Wang Na, Mesfin Haile

**Affiliations:** National Agrobiodiversity Center, National Institute of Agricultural Sciences, Rural Development Administration, Jeonju 54874, Republic of Korea; zzjiy@korea.kr (H.O.); hchko@korea.kr (H.-C.K.); naaeskr@korea.kr (J.Y.); ywna@korea.kr (Y.-W.N.)

**Keywords:** *Capsicum*, fruit-related traits, GWAS, SNPs, pepper breeding

## Abstract

This study utilized 303 pepper accessions from diverse *Capsicum* species to explore fruit traits, including length, width, wall thickness, and weight. Descriptive statistics revealed a mean fruit length of 66.19 mm, width of 23.48 mm, wall thickness of 1.89 mm, and weight of 15.29 g, with significant variability, particularly in fruit weight. Correlation analysis demonstrated strong positive relationships between fruit width, weight, and fruit wall thickness (r = 0.89 and r = 0.86, respectively), while fruit length showed weaker correlations with these traits. Analysis of fruit positions revealed that the majority of accessions had a pendent fruit position (156), followed by erect (85) and intermediate (8). In terms of fruit shape, triangular and narrow triangular shapes were the most common, observed in 102 and 98 accessions, respectively. Genome-wide association studies (GWAS) identified significant single nucleotide polymorphisms (SNPs) associated with fruit traits across four models (Blink, FarmCPU, MLM, MLMM). The number of significantly associated SNPs were as follows: fruit length (89), fruit width (55), fruit weight (63), fruit wall thickness (48), fruit shape (151), and fruit position (51). Several genes were also identified where the SNPs are located or adjacent to, providing candidate genes for further exploration of the genetic basis of fruit morphology. Notably, genes such as E3 ubiquitin-protein ligase RGLG1 (associated with fruit width), Homeobox-leucine zipper protein HDG11 (involved in fruit width), Auxin response factor 23 (linked to fruit shape), and ATP-dependent zinc metalloprotease FtsH (related to fruit weight) were identified. These findings enhance our understanding of the genetic basis of fruit morphology in *Capsicum*, offering valuable insights for breeding and agricultural practices.

## 1. Introduction

The domestication of five major *Capsicum* species (*C. annuum*, *C. baccatum*, *C. chinense*, *C. frutescens*, and *C. pubescens)* occurred roughly 6000 years ago in the Americas [1,2,3]. According to data from FAOSTAT [4], the production of green peppers increased from 31 million tons in 2012 to nearly 37 million tons by 2022. In the same timeframe, production of dry peppers grew from 3.37 million tons to 4.91 million tons. In 2022, China led the world in fresh pepper production, yielding 16.57 million tons, followed by Mexico with 2.73 million tons, Turkey at 2.50 million tons, and Indonesia at 2.29 million tons. India was the top producer of dry peppers, contributing 1.74 million tons to the global market [4].

*Capsicum* species are known for their high content of bioactive compounds, which offer a range of health benefits. These compounds are associated with potential analgesic, anti-obesity, cardioprotective, pharmacological, neurological, and dietary effects [5]. The variety of bioactive compounds present in peppers underscores their importance as a beneficial ingredient in various health applications. Among the primary bioactive components found in *Capsicum* species are carotenoids, capsaicinoids, and vitamins C and E [5].

The genus *Capsicum* encompasses a wide range of fruit traits, including variations in size, shape, weight, and structure, which are of significant interest to both consumers and breeders [6,7]. The agronomic traits of pepper have many categories with abundant phenotypes, which affect the roots, stems, leaves, flowers, and fruits [8,9,10]. Understanding the genetic basis of these trait variations is crucial for developing improved pepper varieties that meet market demands and agricultural needs. In this study, we focused on several key fruit traits: length, width, wall thickness, weight, position, and fruit shape. These traits collectively contribute to the overall fruit quality, yield, and marketability of pepper varieties [11,12]. Several studies have explored the genetic diversity related to fruit size, weight, shape, color, and metabolites within the Solanaceae family, including tomato, eggplant, and pepper. However, the insights into fruit shape and size variation have been mostly confined to the identification of quantitative trait loci (QTL) [11,13,14,15,16,17].

Research focusing on quantitative traits in *Capsicum* spp. has identified the fs3.1 and fs10.1 loci on chromosomes 3 and 10, respectively, as major contributors to fruit shape elongation [18,19]. Similarly, loci like fs8.1 and ovate, which influence fruit shape elongation in tomato [10,20], have been observed at corresponding genomic locations in pepper [21]. For fruit weight, the fw2.2 and fw3.2 QTL in tomato, associated with the cell number regulator (CNR) and KLUH genes, respectively, have also been identified in pepper [13,22]. Correspondingly, fw2.1 and fw4.1 QTL from pepper were mapped to the syntenic positions of the fw2.1 and fw4.2b loci in tomato [21,23]. Additionally, the number of locules (seed-containing cavities derived from carpels), which impacts fruit shape and size, is primarily regulated by the fascinated (fas) and locule number (lc) QTL in tomato [24]. The interplay of these QTL accounts for a significant portion of the variation in locule number [25,26]. While fas and lc loci are critical, other QTL controlling locule number have been reported [27], but the specific genes responsible for these QTL affecting floral meristem development have yet to be identified.

Recent advances in genomic technologies and high-throughput sequencing have revolutionized our ability to study complex traits in crop species [28,29,30]. Genome-Wide Association Studies (GWAS) have emerged as a powerful tool for identifying genetic variants associated with phenotypic diversity in plant populations [30,31]. GWAS have been successfully applied to various crops, including rice [32], maize [33], and tomato [34], revealing key genomic regions linked to agronomically important traits. In pepper, previous studies have primarily relied on traditional QTL mapping and candidate gene approaches to investigate the genetic control of fruit traits [16,19,35]

In this study, a GWAS approach using multiple models was carried out on a pepper core collection to identify genomic regions associated with variations in fruit traits, leveraging the core collection’s genetic diversity and high-density SNP markers. This research aims to enhance understanding of the genetic basis for fruit trait variation in peppers and provide insights for breeding programs focused on developing improved varieties with desirable characteristics, by investigating the relationships among five key fruit-related traits and pinpointing significant genetic regions or genes associated with these traits.

## 2. Results

### 2.1. Fruit Traits of Capsicum

**Figure 1:**
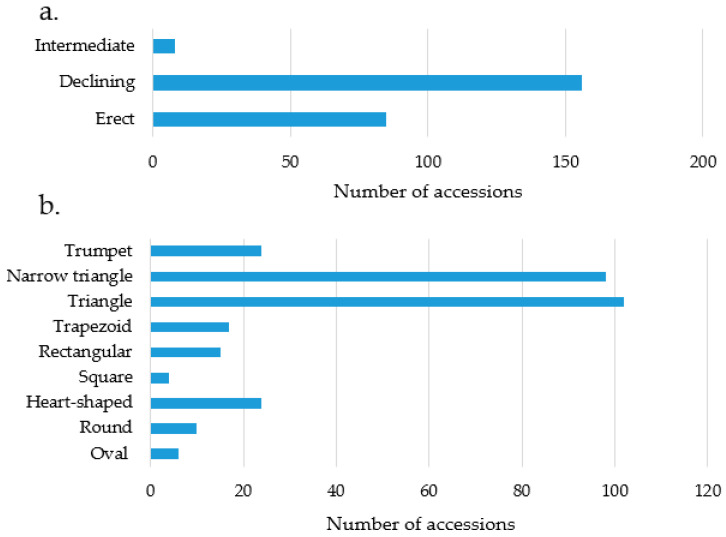
The number of accessions based on the fruit position type (**a**) and fruit shape (**b**) of pepper accessions.

This study revealed substantial diversity in fruit-related traits among the *Capsicum* accessions. The descriptive statistics for these traits are summarized in Table 1. The mean values for fruit length, width, weight, and wall thickness were 66.19 mm, 23.48 mm, 15.29 g, and 1.89 mm, respectively. The ranges observed were 9.27 to 195.93 mm for fruit length, 4.03 to 87.20 mm for fruit width, 0.20 to 165.93 g for fruit weight, and 0.10 to 7.17 mm for fruit wall thickness. The analysis of pepper fruit characteristics revealed considerable variation in both fruit positions and shapes among the accessions. In terms of fruit position, the majority of accessions (156) exhibited a pendent orientation, followed by 85 accessions with an erect position and 8 accessions with an intermediate position (Figure 1a). Fruit shapes also varied significantly, with the most common being triangular, observed in 102 accessions, closely followed by narrow triangular in 98 accessions. Less common shapes included heart-shaped and trumpet (24 accessions each), trapezoid (17), rectangular (15), round (10), oval (6), and square (4) (Figure 1b).

### 2.2. Correlation and Principal Component Analysis

**Figure 2:**
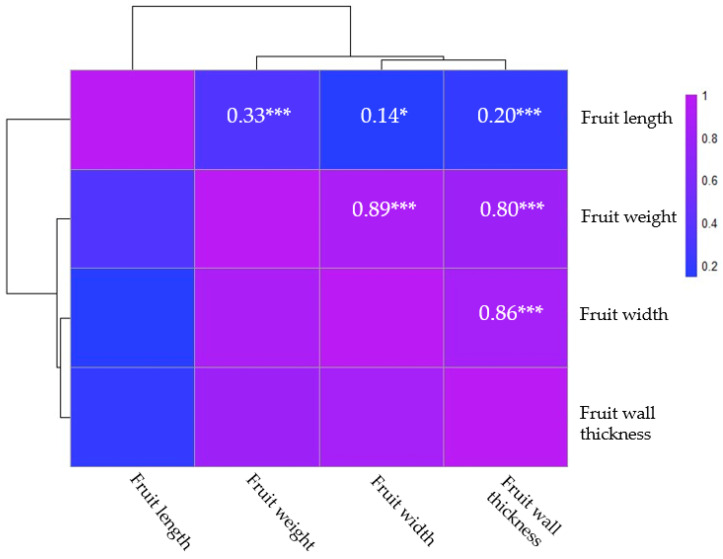
Pearson correlation analysis of fruit-related traits of pepper accessions. Significance is indicated by * and *** for *p*-values of less than 0.05 and 0.001, respectively.

The correlation analysis of the fruit characteristics revealed significant relationships among fruit length, fruit width, fruit weight, and fruit wall thickness (Figure 2). A weak positive correlation was observed between fruit length and fruit width (r = 0.14 *), indicating a slight tendency for longer fruits to also be wider. Similarly, fruit length showed a moderate positive correlation with fruit weight (r = 0.33 ***) and a weak positive correlation with fruit wall thickness (r = 0.20 **), suggesting that longer fruits tend to be somewhat heavier and have slightly thicker walls. In contrast, fruit width exhibited very strong positive correlations with both fruit weight (r = 0.89 ***) and fruit wall thickness (r = 0.86 ***), implying that wider fruits are significantly heavier and have thicker walls. Additionally, a strong positive correlation was found between fruit weight and fruit wall thickness (r = 0.80 ***), indicating that heavier fruits generally possess thicker walls. These findings highlight the intricate relationships among the physical traits of the fruits, providing insights that could be valuable for agricultural practices, quality control, and marketing strategies.

**Figure 3:**
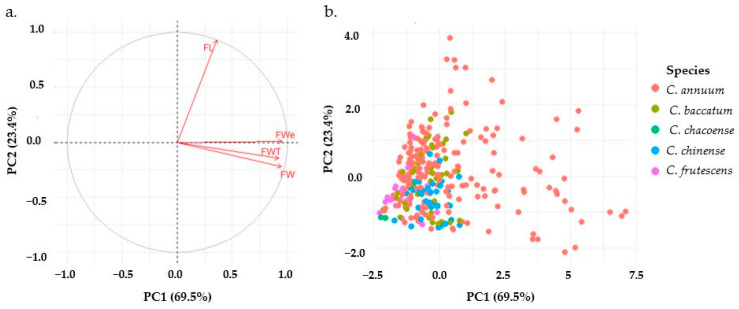
Principal component analysis of fruit related traits of pepper accessions. (**a**): variables, (**b**): individual accessions. FL: fruit length, FW: fruit width, Fwe: fruit weight, FWT: fruit wall thickness.

Principal Component Analysis (PCA) was conducted to elucidate the underlying structure of the fruit morphological data (Figure 3). The first two principal components (PCs) accounted for 92.9% of the total variance, with PC1 and PC2 explaining 69.5% and 23.4% of the variance, respectively. PC1 was strongly positively correlated with fruit width (FW, 0.95), fruit wall thickness (FWT, 0.92), and fruit weight (FWe, 0.95), while fruit length (FL) showed a relatively weak correlation (0.36). In contrast, PC2 was predominantly characterized by fruit length (FL, 0.93). These results suggest that fruit width, wall thickness, and weight vary simultaneously and account for the primary source of variation in the dataset, while fruit length appears to vary independently, representing a secondary, distinct source of morphological variation. This PCA provides insights into the interrelationships among fruit morphological traits, indicating that width, wall thickness, and weight are closely associated, while length varies more independently.

### 2.3. Genome-Wide Association Analysis

**Figure 4:**
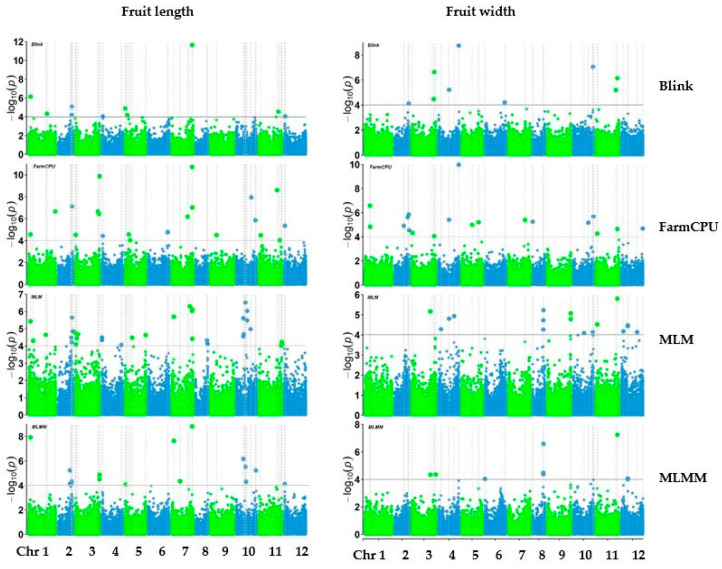
Manhattan plots of fruit length and width of pepper.

**Figure 5:**
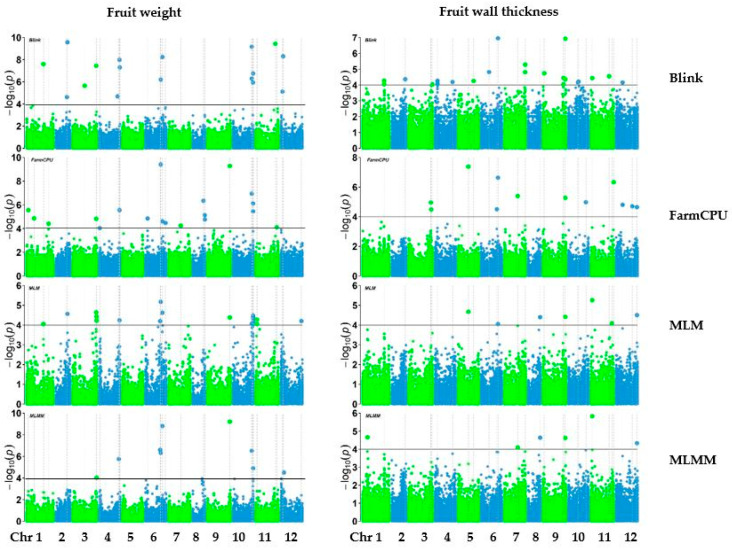
Manhattan plots of fruit weight and fruit wall thickness.

**Figure 6:**
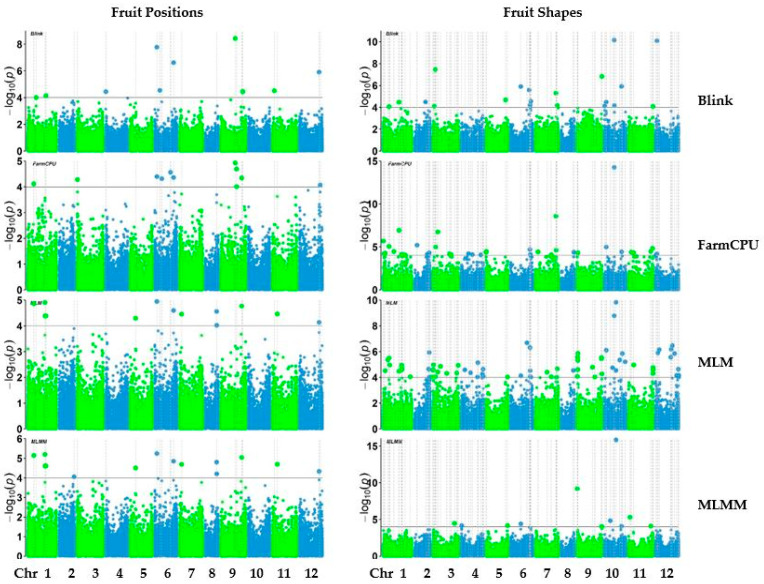
Manhattan plots of fruit position and shape of pepper.

**Figure 7:**
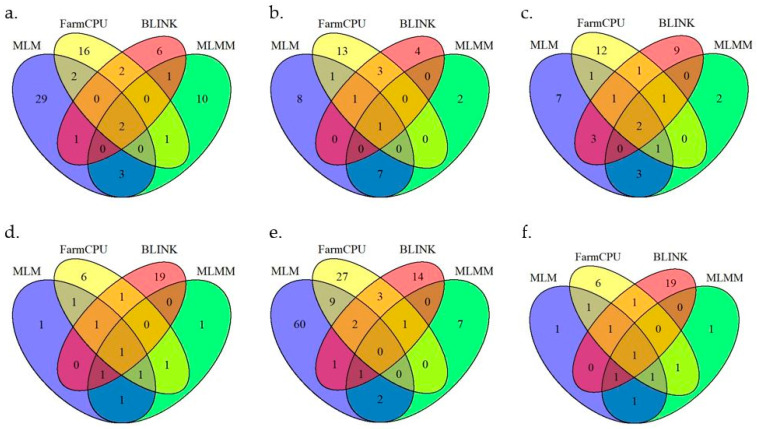
Venn diagram showing shared and unique numbers of significantly associated SNPs with fruit-related traits across multiple models. (**a**): fruit length, (**b**): fruit width, (**c**): fruit weight, (**d**): fruit wall thickness, (**e**): fruit shape, (**f**): fruit position.

Genome-wide association analysis was conducted with 38,079 SNPs generated from 303 pepper accessions. The number of SNPs significantly associated with each trait varied across the models (Blink, FarmCPU, MLM, and MLMM) (Figure 4, Figure 5 and Figure 6). The GWAS results are visualized in manhattan plots (Figure 4, Figure 5 and Figure 6) and quantile–quantile plots (Q-Q) (Supplementary Figure S1). The shared and unique numbers of significantly associated SNPs with fruit-related traits across multiple models are visualized via Venn diagram (Figure 7). For fruit length, the highest number of significant SNPs were identified: Blink with 12, FarmCPU with 23, MLM with 37, and MLMM with 17. For fruit width, the models detected 9 SNPs with Blink, 19 SNPs with both FarmCPU and MLM, and 9 SNPs with MLMM. Fruit weight had 17 SNPs identified by Blink, 19 by FarmCPU, 18 by MLM, and 9 by MLMM. For fruit wall thickness, Blink identified the most SNPs with 23, followed by FarmCPU with 12, MLM with 7, and MLMM with 6. Fruit shape had the highest count overall: Blink identified 22 SNPs, FarmCPU 42, MLM 75, and MLMM 11. Lastly, for fruit positions, the numbers were 12 SNPs by Blink, 11 by FarmCPU, 13 by MLM, and 14 by MLMM. A list of selected SNPs associated with fruit-related traits are presented in Table 2. All SNPs associated with fruit traits using all four models are presented in Supplementary Table S1.

Among the SNPs significantly associated with fruit length, key genes identified include S03_277572673 on chromosome 3, associated with the glycosyltransferase 61 catalytic domain-containing protein (FarmCPU); S03_282615360 on chromosome 3, linked to the Mediator of RNA polymerase II transcription subunits 7 and 8 (FarmCPU and MLMM); S10_165832202 on chromosome 10, related to B3 domain-containing transcription repressor VAL2 (MLM); S02_157086752 on chromosome 2, associated with eukaryotic translation initiation factor 5 (Blink and MLM); and S07_240926377 on chromosome 7, linked to phosphoenolpyruvate carboxylase 2 (FarmCPU and MLM). For fruit width, significant SNPs include S04_121266310 on chromosome 4, showing strong associations with ATP-dependent zinc metalloprotease FtsH (FarmCPU, Blink, and MLM); S10_173068502 on chromosome 10, related to ammonium transporter 2 member 2; and S02_167970112 on chromosome 2, linked to E3 ubiquitin-protein ligase RGLG1. Other notable SNPs include S01_56406344 for E3 ubiquitin-protein ligase UPL6, S03_257466169 for homeobox-leucine zipper protein HDG11, and S12_23139281 for putative pectinesterase/pectinesterase inhibitor 32.

Significant SNPs associated with fruit weight include S06_197169464 on chromosome 6, closely downstream of the 18 kDa seed maturation protein (Blink, FarmCPU, MLM, MLMM); S10_217430668 on chromosome 10, associated with ABC transporter F family members (Blink, FarmCPU, and MLMM); and S10_231729482 on chromosome 10, linked to L-galactono-1,4-lactone dehydrogenase (FarmCPU). Additionally, S01_18308379 on chromosome 1, associated with protein NRT1/PTR FAMILY, and S03_274011164 on chromosome 3, related to putative cyclic nucleotide-gated ion channel 9, are significant. S01_195688472 on chromosome 1, linked to putative WRKY transcription factor 69, S12_238461338 on chromosome 12, associated with RING-type E3 ubiquitin transferase, and S06_233976320 on chromosome 6, linked to UDP-D-apiose/UDP-D-xylose synthase 2.

For fruit wall thickness, key SNPs include S06_197169464 on chromosome 6, which is linked to the 18 kDa seed maturation protein (identified by Blink, FarmCPU, and MLM); S11_246196612 on chromosome 11, associated with the AB hydrolase-1 domain-containing protein (MLM); and S09_262305652 on chromosome 9, related to the Arf-GAP domain-containing protein (Blink). Additionally, S06_184463863 on chromosome 6 is linked to the C2 domain-containing protein (FarmCPU), while S12_242888277 on chromosome 12 is associated with various disease resistance proteins (FarmCPU, MLM, and MLMM). Other significant SNPs include S05_180422718 and S05_180422776 on chromosome 5, associated with Fe_2_OG dioxygenase domain-containing protein (Blink), and S07_245029775 on chromosome 7, linked to formin-like protein 5 (Blink), both located close downstream of these genes.

For fruit wall thickness, key SNPs include S06_197169464 on chromosome 6, linked to the 18 kDa seed maturation protein (Blink, FarmCPU and MLM); S11_246196612 on chromosome 11, associated with AB hydrolase-1 domain-containing protein (MLM); and S09_262305652 on chromosome 9, related to Arf-GAP domain-containing protein (Blink). Also significant are S06_184463863 on chromosome 6, associated with C2 domain-containing protein (FarmCPU), and S12_242888277 on chromosome 12, linked to various disease resistance proteins (FarmCPU, MLM, and MLMM). S05_180422718 and S05_180422776 on chromosome 5, associated with Fe_2_OG dioxygenase domain-containing protein (Blink), and S07_245029775 on chromosome 7, related to formin-like protein 5 (Blink), are located closely downstream of these genes.

For fruit shape, S09_3082202 on chromosome 9, located very close to the downstream gene DnaJ protein ERDJ2A, is strongly associated with fruit shape (*p* = 6.42 × 10^−10^). Additional significant SNPs include S05_760811 on chromosome 5, linked to calmodulin-binding domain-containing protein; S05_2981666 on chromosome 5, associated with subtilisin-like protease SBT5.3; and S01_61467129 on chromosome 1, related to ethylene insensitive 3-like DNA-binding domain-containing protein. Furthermore, S10_63295730 on chromosome 10, associated with protein trichome birefringence-like 25, and S07_225971548 on chromosome 7, linked to ethylene-responsive transcription factor 4, contribute to the genetic basis of fruit shape variation.

For fruit position, SNP S09_156164798 on chromosome 9, associated with the regulatory-associated protein of TOR 1, is the most significant. Other significant SNPs include S06_204745890 on chromosome 6, linked to T-complex protein 1 subunit eta, and S01_63889989 on chromosome 1, very close to K homology domain-containing protein. Additionally, S09_227011022 on chromosome 9, associated with prolyl endopeptidase, and S12_226143954 on chromosome 12, near subtilisin-like protease SBT1.9, are also significant. These SNPs are positioned near but not directly on these genes, suggesting they might play a role in determining fruit position.

## 3. Discussion

The considerable diversity observed in *Capsicum* fruit traits in this study highlights the genetic richness within the genus. Peppers exhibit significant variation in various agronomic characteristics, including fruit-related traits [14,36,37,38,39]. This study also observed notable variations in pepper fruit length, width, weight, wall thickness, position, and shape. These fruit-related traits are critical determinants of quality. This diversity is crucial for breeding programs and genetic resource management, offering a wide range of traits for selection and improvement. With advancements in high-throughput sequencing technology, GWAS have emerged as a powerful tool for identifying candidate genes associated with various traits [40]. In our study, GWAS using multiple models were conducted on six fruit-related traits of peppers, revealing significant SNPs associated with these traits. The use of multiple models helped us to explore more possible candidate genes. Several SNPs associated with the fruit-related traits that are located on several genes are discussed below. Understanding the genetic basis of these traits is critical, as cell number and size are pivotal determinants of plant organ size, and any variation in these parameters can impact organ size significantly [41].

Based on our finding of an SNP associated with fruit length in *Capsicum* in a gene encoding phosphoenolpyruvate carboxylase 2 (PEPC2), we can draw important parallels with existing literature on PEPC’s role in fruit development across various species. PEPC has been shown to play a crucial role in fruit growth, ripening, and organic acid metabolism in several fruits, including tomato, peach, and grape [42,43,44]. The enzyme catalyzes the carboxylation of phosphoenolpyruvate to oxaloacetate, a key step in organic acid synthesis and carbon fixation [42,45]. In tomato and peach, PEPC activity and expression increase during fruit development, peaking at the onset of ripening, suggesting its importance in critical stages of fruit growth [42,43]. Furthermore, studies on tomato have shown that PEPC deficiency affects fruit sugar content and overall plant growth [46], highlighting its significance in fruit metabolism. The phosphorylation of PEPC, as observed in grape, tomato, cherry, and plum [47], suggests a potential regulatory mechanism that could fine-tune its activity in coordination with other enzymes like PEPCK. Our finding of an SNP in PEPC2 associated with fruit length in pepper aligns with these observations, suggesting that variations in PEPC2 might affect carbon metabolism, organic acid synthesis, or cell expansion in *Capsicum* fruits, thereby influencing fruit length. This connection between PEPC2 and fruit size opens up new avenues for understanding the molecular mechanisms underlying fruit development in *Capsicum* and could have potential applications in breeding programs aimed at modifying fruit characteristics and quality traits such as sugar–acid balance.

Among the SNPs associated with fruit width, several genes encode notable candidate proteins, including AB hydrolase-1 domain-containing protein, E3 ubiquitin-protein ligase RGLG1, homeobox-leucine zipper protein HDG11, ATP-dependent zinc metalloprotease FtsH, putative pectinesterase/pectinesterase inhibitor 32, transcription factors PCF2 and PCF3, and sarcoplasmic/endoplasmic reticulum calcium ATPase 3. These candidates are significant for further investigation due to their established roles in growth regulation, cell expansion, and cell wall modification. In particular, E3 ubiquitin-protein ligase RGLG1 stands out due to its potential involvement in regulating plant hormone signaling. This aligns with previous studies, notably on the role of RGLG5, a close homolog of RGLG1, which mediates the degradation of PP2CA, a phosphatase that inhibits ABA signaling [48]. Recent findings from a high-resolution genome-wide association study have also implicated RGLG5 in fruit size and weight, as it was identified in a haplotype block surrounding the significant SNP marker chr1_33914270, which is associated with fruit width and weight in sweet cherry (*Prunus avium* L.) [49]. Both RGLG1 and RGLG5 are involved in controlling levels of ABA [48], a key hormone regulating fruit ripening and size. The synchronization of auxins, gibberellins, and ABA levels is critical during fruit development, with ABA being suppressed during early stages of fruit growth and then accumulating at the onset of ripening [50]. Given the similar roles of RGLG1 and RGLG5 in ABA regulation, RGLG1 becomes a strong candidate for influencing fruit size by modulating ABA levels during fruit development. Additionally, homeodomain-leucine zipper (HD-ZIP) proteins participate in regulating plant growth and development, including fruit development and maturity, anthocyanin accumulation, flowering, vascular development, and epidermal cell development [51]. Specifically, HD-ZIP proteins regulate fruit ripening by modulating cell wall degradation and ethylene biosynthesis [51]. In banana (*Musa acuminata*), the HD-ZIP I genes MaHDZI.19 and MaHDZI.26, along with the HD-ZIP II genes MaHDZII.4 and MaHDZII.7, were significantly upregulated during fruit ripening [51]. These four MaHDZs localize in the nucleus and activate several maturation-related genes, including MaACO5, related to ethylene biosynthesis, and MaEXP2, MaEXPA10, MaPG4, and MaPL4, which are associated with cell wall degradation [52]. Similarly, LcHB2, a member of the litchi (*Litchi chinensis*) HD-ZIP I subfamily, regulates fruit drop by directly activating cell wall degradation-related genes LcCEL2 and LcCEL8, while the HD-ZIP I gene LcHB3 promotes ethylene biosynthesis [53,54]. The HD-ZIP II gene PpHB.G7 interacts with the promoters of the ethylene biosynthesis genes PpACS1 and PpACO1 to enhance peach (*Prunus persica*) maturation by promoting ethylene production [55]. Furthermore, silencing of MdHB1 in apple results in anthocyanin accumulation by releasing transcription factors MdTTG1, MdMYB10, and MdbHLH3 from the cytoplasm, leading to the activation of MdDFR and MdUFGT and promoting red-fleshed apple fruit [56]. TCP transcription factors (TFs), a plant-specific protein family, play significant roles in regulating plant growth and development, including reproductive processes such as flowering time, inflorescence stem growth, and flower organ development [57]. Members of the TCP family have been implicated in various aspects of reproductive development, which may influence traits related to fruit size and maturity. Understanding the functions of TCPs could provide further insights into the molecular networks involved in fruit development, complementing the roles of the other candidate genes identified in our study.

Some of the significantly associated SNPs linked to fruit weight are located in genes encoding ABC transporter F family member 4, cytokinesis protein sepA-like, diphosphomevalonate decarboxylase, L-galactono-1,4-lactone dehydrogenase (mitochondrial), and RING-type E3 ubiquitin transferase. Additionally, there are SNPs close to downstream genes such as the 18 kDa seed maturation protein and the putative WRKY transcription factor 69. ATP-binding cassette (ABC) transporters play crucial roles in transporting a wide range of molecules, including secondary metabolites [58,59], heavy metals for detoxification [60], antibiotics [61], and phytohormones [62,63]. Given these diverse functions, ABC transporters are indispensable for the growth and development of tomato plants, including fruit development [64]. Most ABC transporters contain transmembrane domains (TMDs) and are part of the ABC protein family, which also includes soluble ABC proteins that lack TMDs [64]. The identification of SNPs in genes encoding RING-type E3 ubiquitin ligases associated with pepper fruit weight adds a new dimension to our understanding of fruit size regulation. While much of the recent research has focused on the role of E3 ligases in fruit ripening, our findings suggest these enzymes may also play a critical role in determining fruit size in peppers. This aligns with the diverse functions of E3 ligases observed across various fruit species. For instance, in banana, MaXB3 and MaEBF1 [65,66], as well as Sl-EBF1/EBF2 in tomato [67], have been shown to regulate fruit ripening processes. Similarly, E3 ligases like MaBRG2/3 in banana [67] and MdPUB29 in apple [68] control ripening by modulating the stability of ripening-related transcription factors. While these studies primarily focus on ripening, the underlying mechanisms of protein degradation and transcription factor regulation could also be relevant to fruit size determination. The SNPs we identified in pepper E3 ligase genes might affect similar regulatory pathways, potentially influencing cell division, cell expansion, or other processes that contribute to final fruit weight. Moreover, the proximity of identified SNPs to downstream genes such as the 18 kDa seed maturation protein and the putative WRKY transcription factor 69 suggests potential regulatory roles in fruit development. WRKY transcription factors are known to play important roles in plant growth and stress responses, which are critical for optimal fruit development and weight.

An SNP associated with fruit wall thickness was identified in a gene encoding formin-like protein 5. Formins are conserved actin polymerization machines that play crucial roles in controlling actin cytoskeleton rearrangements and have recently been shown to directly regulate microtubule dynamics [69]. Actin, a highly conserved 42 kDa protein, is abundant in eukaryotes and involved in numerous cellular processes in plants, including cell growth, cell division, cytokinesis, and various intracellular trafficking events [70]. Consequently, actin is essential for plant growth and development [71]. Another SNP associated with fruit wall thickness was found in a gene that encodes hydroxyproline O-arabinosyltransferases. Recent studies have illuminated the critical role of cell wall modifications in plant responses to various stresses and their broader implications for structural traits. Hydroxyproline O-arabinosyltransferases (HPAT1-3), including HPAT3, are responsible for the initial step of adding arabinose residues to hydroxyproline (Hyp) residues of extensins, which are crucial for cell wall integrity [72]. These enzymes facilitate the foundational step in the arabinosylation of extensins, essential for their subsequent crosslinking and strengthening of the cell wall matrix. A study identified extensin arabinose deficient transferase (ExAD) as a key gene affecting root cell wall thickness under salt stress conditions, where mutants lacking the final arabinose addition to extensins displayed increased cell wall thickness and porosity [73]. This highlights the importance of proper arabinosylation, facilitated by enzymes like HPAT3, in maintaining optimal cell wall properties. The identification of an SNP in the HPAT3 gene associated with fruit wall thickness in *Capsicum* aligns with the established role of hydroxyproline O-arabinosylation in cell wall integrity and modification. The involvement of HPAT3 in cell wall architecture [72] suggests its significant impact on fruit wall properties, making it a key gene for further research and breeding programs aimed at improving fruit quality traits.

In this study, we identified an SNP associated with fruit shape in *Capsicum*, located in the gene encoding auxin response factor 23 (ARF23). This discovery is significant given the well-established role of ARFs in regulating various aspects of fruit development through the auxin signaling pathway. Auxin is a key phytohormone that plays a critical role in controlling fleshy fruit development and ripening through its signaling pathway [74]. The process involves auxin binding to the TIR1/AFB receptors, which then recruit other components to form an SCF ubiquitin ligase complex, leading to the degradation of inhibitory Aux/IAA proteins. This degradation releases ARFs, which then regulate auxin-dependent gene expression [75,76]. ARFs are integral to this process, linking auxin signaling to downstream responses that govern fruit development and shape [77,78]. Previous studies have demonstrated the involvement of ARFs in fruit development across multiple species, including tomato and papaya, where specific ARF genes have been linked to fruit set and ripening [79,80,81]. In particular, in papaya, several CpARF genes, including CpARF2, CpARF6, CpARF7, CpARF10, CpARF16, and CpARF17, displayed fruit-specific expression patterns, suggesting their importance in improving fruit-related agronomic traits in papaya [82]. CpARF6 expression increased during the developmental process and reached its peak level at the final stage of flower development [83]. The expression of CpARF1 increased significantly during the fruit ripening stages [83]. Many AuxREs were included in the promoters of two ethylene signaling genes (CpETR1 and CpETR2) and three ethylene synthesis-related genes (CpACS1, CpACS2, and CpACO1), suggesting that CpARFs might be involved in fruit ripening via the regulation of ethylene signaling [83]. Moreover, the interaction between auxin and ethylene signaling, mediated by ARFs, further highlights their importance in fruit morphology and quality.

Among the SNPs significantly associated with fruit positions are those located in a gene that encodes regulatory-associated protein of TOR, putative WRKY transcription factor 69, T-complex protein 1 subunit eta, vesicle transport protein and fidgetin-like protein. Target of Rapamycin (TOR) is a positive regulator of growth and development in all eukaryotes, which positively regulates anabolic processes like protein synthesis, while repressing catabolic processes including autophagy [84]. As a consequence, this work indicates that a functional TOR signaling pathway is not only highly relevant in the process of seed germination and metabolism, but is also required for the proper development of the mature seed [84]. The WRKY transcription factor family is a key player in the regulatory mechanisms of flowering plants, significantly influencing both their biotic and abiotic response systems as well as being vital to numerous physiological and biological functions.

In conclusion, this study enhances our understanding of the genetic diversity and molecular mechanisms influencing fruit traits in *Capsicum* species. The significant SNPs identified through GWAS, associated with key genes involved in growth regulation and hormone signaling, underscore the intricate relationships between genetic factors and fruit characteristics. However, further investigation is crucial to explore the functional roles of these genes and their interactions within broader genetic networks, which could inform targeted breeding strategies to improve fruit quality and yield.

## 4. Materials and Methods

### 4.1. Plant Materials

The study comprised 303 pepper accessions provided by the gene bank of the National Agrobiodiversity Center (NAC), under the Rural Development Administration (RDA) in Jeonju, Republic of Korea. These accessions are from five species: *C. annuum* (195 accessions), *C. baccatum* (44), *C. chinense* (41), *C. frutescens* (21), and *C. chacoense* (2). The plants were cultivated in a greenhouse at the RDA research field (Jeonju, Republic of Korea) (35°49′52.7″ N, 127°3′43.9″ E) from March to October 2020, following standard agronomic practices as per RDA guidelines. Seedlings were prepared between March and April, with temperatures controlled between 15 and 25 °C. In May, the seedlings were transplanted to the soil and grown until late October, with the greenhouse temperature kept between 15 and 40 °C. Irrigation was carried out once or twice a week based on the plants’ needs. Fertilization was conducted according to soil analysis results and RDA’s standard methods, which included base fertilization amounts of 16.4 kg nitrogen, 16.8 kg phosphorus, and 16.8 kg potassium per 10 Ares (a), as well as additional fertilization amounts of 13.4 kg nitrogen and 11.2 kg potassium per 10 a. During cultivation, the greenhouse sides and doors were opened to mimic the humidity and light conditions typical of the Jeonju region in spring to fall. Each accession was represented by ten pepper plants. Further details about the accession numbers and origins of the 306 accessions can be found in Supplementary Table S2.

### 4.2. Pepper Fruit Trait Assessment

Ten plants per accession were used for phenotypic evaluation. Six fruit-related traits, including four quantitative traits (fruit length, width, weight, and fruit wall thickness) and two qualitative traits (fruit shape and fruit position), were assessed. All quantitative traits were measured using a digital caliper. Fruit position was scored on a scale of 1 to 3 (1 = erect, 2 = pendent, 3 = intermediate). After harvest, the vertically cut surface of the fruit was examined, and its shape was characterized by referring to the reference images (Supplementary Figure S2). The shape was evaluated on a scale of 1 to 9 (1 = oval (laying horizontally), 2 = round, 3 = heart-shaped, 4 = square, 5 = rectangular, 6 = trapezoid, 7 = triangle, 8 = narrow triangle, 9 = trumpet).

### 4.3. Genomic DNA (gDNA) Extraction

Genomic DNA was extracted from leaf tissues of 303 pepper accessions using a modified CTAB method as described by Lee et al. [85]. The extracted DNA was diluted to a concentration of 50 ng/μL with distilled water. DNA quantification was conducted using the Quant-iT PicoGreen dsDNA Assay Kit (Thermo Fisher Scientific, Waltham, MA, USA) and a Synergy HTX Multi-Mode Reader (BioTek, Instruments, Winooski, VT, USA). Afterward, DNA concentrations were adjusted to 12.5 ng/μL. The standardized DNA samples were then digested with the ApeKI restriction enzyme (New England Biolabs, Ipswich, MA, USA) at 75 °C for 3 h.

### 4.4. Library Preparation for Genotyping-by-Sequencing (GBS)

GBS libraries were prepared following the methods of [86,87], with minor modifications. After restriction digestion, DNA fragments were ligated with adapters, including barcoded adapters for sample identification and common adapters, using T4 DNA ligase (New England Biolabs, Ipswich, MA, USA) at 22 °C for 2 h. The ligase was then inactivated by heating at 65 °C for 20 min. The adapter-ligated samples were pooled and purified using the NucleoSpin^®^ Gel and PCR Clean-up kit (Macherey-Nagel GmbH & Co. KG, Düren, Nordrhein-Westfalen, Germany). The pooled ligation products were amplified by multiplex PCR in a 50 μL reaction volume using AccuPower Pfu PCR Premix (Bioneer, Daejeon, Republic of Korea) and the provided primers. The fragment size distribution of the PCR products was assessed using the BioAnalyzer 2100 (Agilent Technologies, Santa Clara, CA, USA). Finally, the GBS libraries were sequenced on the Illumina NextSeq500 platform (Illumina, San Diego, CA, USA), generating 150 bp single-end reads.

### 4.5. SNP Calling, Filtering, and Sequence Preprocessing

The read sequences generated were preprocessed through several steps: Stacks [88] was used for demultiplexing, FastQC [89] for assessing per-base read quality, and Cutadapt [90] for trimming adapter sequences. The reads were subsequently aligned to the CM334 reference genome (*C. annuum* chromosome v1.6) using Bowtie2. To facilitate integration into the GATK pipeline, read groups were incorporated using Picard tools. Localized realignment of reads was carried out with Genome Analysis Toolkit (GATK) ‘IndelRealigner’ and ‘RealignerTargetCreator’ functions to correct misalignments due to indels.

Initial variant calling was performed using GATK’s “HaplotypeCaller” and “SelectVariants” parameters. Variants were then filtered with GATK’s “FilterVariant” module based on a quality score (QUAL < 30), quality depth (QD < 5), and Fisher score (FS > 200). Additional filtering was done with vcftools (v. 0.1.15) to set restrictions on the maximum missing rate (--max-missing 0.95), minimum minor allele frequency (--maf 0.05), allele range (--min-alleles 2, --max-alleles 2), and average read depth (--min-meanDP 5). These steps were taken to identify high-quality SNPs for subsequent analysis.

### 4.6. Genome-Wide Association Study (GWAS)

A genome-wide association study (GWAS) was performed on 303 pepper individuals using 38,079 single nucleotide polymorphisms (SNPs). The analysis utilized the R-based Genome Association and Prediction Integrated Tool (GAPIT version 3). Four models were employed: mixed linear model (MLM) [91], Multiple Loci Mixed Linear Model (MLMM) [92], Fixed and Random Model Circulating Probability Unification (FarmCPU) [93], and Bayesian-information and Linkage-disequilibrium Iteratively Nested Keyway (BLINK) [94]. All models incorporated population structure and kinship (PCA + K). The kinship matrix was derived from an identical-by-state (IBS) matrix, reflecting genetic relationships between lines. Significance was set at −log_10_ (10^−5^) > 4.0.

To identify potential candidate genes, a 200-kb region surrounding each significant SNP was examined. The Basic Local Alignment Search Tool (BLAST) was used to search the *C. annuum* genome in both Ensemble Plants (https://plants.ensembl.org/Capsicum_annuum/Info/Index accessed on 17 June 2024) and National Center for Biotechnology Information (NCBI) databases (https://blast.ncbi.nlm.nih.gov/Blast.cgi accessed on 17 June 2024). Flanking sequences of significant SNPs were analyzed to find functionally similar genes or regions.

### 4.7. Statistical Analysis

Data analysis was conducted using Microsoft Excel 2021 for basic summaries and descriptive statistics of fruit-related data. R software (version 4.2.1) was used for more advanced analyses. These included Pearson correlation analysis using the “pheatmap” package, PCA (FactoMineR package) and GWAS (GAPIT version 3).

## Figures and Tables

**Table 1:** Descriptive statistics of fruit-related characteristics of pepper.

Variable	Fruit Length	Fruit Width	Fruit Weight	Fruit Wall Thickness
Mean	66.19	23.48	15.29	1.89
SE	1.94	0.90	1.34	0.07
SD	33.76	15.63	23.25	1.15
Minimum	9.27	4.03	0.20	0.10
Maximum	195.93	87.20	165.93	7.17
Count	303	303	303	303

Note: SD—standard deviation; SE—standard error.

**Table 2:** List of selected SNPs associated with fruit-related traits.

Traits	SNP	Chr.	Pos. (bp)	−Log (*p*-Value)	Models	Gene Descriptions
Fruit length	S04_143118	4	143118	4.49	MLM	Acylamino-acid-releasing enzyme
	S04_143047	4	143047	4.34		
	S10_165832202	10	165832202	4.98	MLM	B3 domain-containing transcription repressor VAL2
	S02_157086752	2	157086751	4.22, 4.19, 4.22	MLM, Blink, MLM	Eukaryotic translation initiation factor 5
	S03_277572673	3	277572673	6.43	FarmCPU	Glycosyltransferase 61 catalytic domain-containing protein
	S03_282615215	3	282615215	4.50	MLMM	Mediator of RNA polymerase II transcription subunit 6
	S03_282615360	3	282615360	9.87, 4.87	FarmCPU, MLMM	
	S07_240926377	7	240926377	7.02, 6.11	FarmCPU, MLM	Phosphoenolpyruvate carboxylase 2
	S02_158455020	2	158455020	4.32, 4.21	MLMM, Blink	Protein kinase domain-containing protein (Upstream)
	S01_304887691	1	304887691	6.66	FarmCPU	Protein transport protein sec16
	S03_1849591	3	1849591	4.76	MLM	
	S03_8074443	3	8074443	4.53	FarmCPU	putative pectate lyase 9
	S01_195710201	1	195710201	4.65	MLM	putative WRKY transcription factor 69
	S02_154077007	2	154077007	4.50	MLM	Transcription initiation factor TFIID subunit 14b
	S07_240937887	7	240937887	4.41	MLM	UDP-N-acetylglucosamine 1-carboxyvinyltransferase
Fruit Width	S10_173068502	10	173068502	5.18	FarmCPU	Ammonium transporter 2 member 2
	S04_121266310	4	121266310	5.42, 5.22, 4.81	FarmCPU, Blink, MLM	ATP-dependent zinc metalloprotease FtsH
	S02_167970112	2	167970112	4.55	FarmCPU	E3 ubiquitin-protein ligase RGLG1
	S01_56406344	1	56406344	4.84	FarmCPU	E3 ubiquitin-protein ligase UPL6
	S03_257466169	3	257466169	4.48	Blink	Homeobox-leucine zipper protein HDG11
	S12_23139281	12	23139281	4.17	MLM	putative pectinesterase/pectinesterase inhibitor 32
	S11_230838820	11	230838820	5.20	Blink	Putative pentatricopeptide repeat-containing protein
	S10_226033753	10	226033753	4.14	MLM	Sarcoplasmic/endoplasmic reticulum calcium ATPase 3
	S03_217432070	3	217432070	5.17, 4.34	MLM	Transcription factor PCF2
	S04_28350549	4	28350549	4.28	MLM	Vacuolar protein sorting-associated protein 8 central domain-containing protein
Fruit Weight	S06_197169464	6	197169464	8.24, 4.61, 4.62, 8.80	Blink, FarmCPU, MLM, MLMM	18 kDa seed maturation protein (downstream)
	S10_217430668	10	217430668	9.17, 6.95, 6.52	Blink, FarmCPU, MLMM	ABC transporter F family member 4
	S11_16194037	11	16194037	4.05	MLM	Cytokinesis protein sepA-like
	S12_25680491	12	25680491	8.31	Blink	Diphosphomevalonate decarboxylase
	S10_231729482	10	231729482	6.11	FarmCPU	L-galactono-1,4-lactone dehydrogenase, mitochondrial (upstream)
	S01_18308379	1	18308379	5.56	FarmCPU	Protein NRT1/ PTR FAMILY 7.3
	S03_274011164	3	274011164	4.63	MLM	putative cyclic nucleotide-gated ion channel 9
	S01_195688472	1	195688472	4.05	MLM	putative WRKY transcription factor 69 (downstream)
	S12_238461338	12	238461338	4.20	MLM	RING-type E3 ubiquitin transferase
	S06_233976320	6	233976320	4.48	FarmCPU	UDP-D-apiose/UDP-D-xylose synthase 2 (upstream)
Fruit wall thickness	S06_197169464	6	197169464	6.94, 6.63, 4.05	Blink, FarmCPU, MLM	18 kDa seed maturation protein
	S11_246196612	11	246196612	4.09	MLM	AB hydrolase-1 domain-containing protein
	S09_262305652	9	262305652	4.35	Blink	Arf-GAP domain-containing protein
	S06_184463863	6	184463863	4.50	FarmCPU	C2 domain-containing protein
	S06_92643241	6	92643241	4.81	Blink	Chromosome transmission fidelity protein 8 homolog
	S12_242888277	12	242888277	4.65, 4.50, 4.33	FarmCPU, MLM, MLMM	Disease resistance protein At1g50180
	S05_180422718	5	180422718	4.27	Blink	Fe_2_OG dioxygenase domain-containing protein (downstream)
	S07_245029775	7	245029775	4.81	Blink	Formin-like protein 5 (close to downstream)
	S10_109872456	10	109872456	4.16	Blink	Hydroxyproline O-arabinosyltransferase 3
	S11_212504568	11	212504568	4.55	Blink	Mitochondrial fission 1 protein B
	S04_203010201	4	203010201	4.19	Blink	Sugar transport protein 1
Fruit Shape	S05_760811	5	760811	4.38	FarmCPU	Calmodulin-binding domain-containing protein
	S12_38450261	12	38450261	6.16	MLM	E2F transcription factor-like E2FE
	S01_61467129	1	61467129	5.51, 5.07, 4.07	MLM, FarmCPU, Blink	Ethylene insensitive 3-like DNA-binding domain-containing protein
	S10_63295730	10	63295730	4.83	MLMM	Protein trichome birefringence-like 25
	S04_120057642	4	120057642	4.10	FarmCPU	Transcription factor BIM3
	S10_165832202	10	165832202	5.35	MLM	B3 domain-containing transcription repressor VAL2
	S01_168895985	1	168895985	4.47, 6.92, 4.48	MLM, FarmCPU, Blink	Zinc finger CCCH domain-containing protein 14
	S07_172197834	7	172197834	4.07, 4.04	FarmCPU, MLM	putative receptor-like protein kinase
	S09_194589098	9	194589098	4.03	MLM	Leucine-rich repeat receptor-like protein kinase PXC2
	S10_225216004	10	225216004	5.21	MLM	Calcium-dependent protein kinase 5
	S07_225971548	7	225971548	4.62	FarmCPU	Ethylene-responsive transcription factor 4
	S05_233242376	5	233242376	4.03	MLM	Kinesin-like protein KIF2C
	S07_235536939	7	235536939	4.18	Blink	Serine/threonine-protein phosphatase 5
	S12_250440434	12	250440434	4.16	MLM	Pentatricopeptide repeat-containing protein
	S03_276692305	3	276692305	4.93	MLM	Auxin response factor 23
	S01_292004974	1	292004974	4.05	MLM	Ferredoxin-dependent glutamate synthase
Fruit Positions	S01_63889989	1	63889989	4.12, 4.86, 5.14	FarmCPU, MLM, MLMM	K Homology domain-containing protein (upper stream)
	S06_55281891	6	55281891	4.55	Blink	Lipoyl-binding domain-containing protein
	S02_164411964	2	164411964	4.06	MLMM	Peptide deformylase 1B, chloroplastic
	S01_195710201	1	195710201	4.15, 4.39, 4.61	Blink, MLM, MLMM	putative WRKY transcription factor 69
	S09_156164798	9	156164798	8.43, 4.93	Blink, FarmCPU	Regulatory-associated protein of TOR 1
	S12_226143954	12	226143954	4.07	FarmCPU	Subtilisin-like protease SBT1.9 (Upper stream)
	S06_204745890	6	204745890	6.62, 4.36, 4.60, 4.85	Blink, FarmCPU, MLM, MLMM	T-complex protein 1 subunit eta
	S05_57950417	5	57950417	4.29, 4.51	MLM, MLMM	Vesicle transport protein

Note: SNP = single nucleotide polymorphism, Chr. = Chromosome, Pos. (bp) = Position in base pairs, −Log (*p*-value) = negative logarithm of the *p*-value. The *p*-values and models are presented in respective order.

## Data Availability

Relevant data are included in both the manuscript and the Supplementary Files.

## Supplementary Materials

The following supporting information can be downloaded at: https://www.mdpi.com/article/10.3390/ijms252111836/s1.
