# Supplementary material for: Exploring Genomic Regions Associated with Fruit Traits in Pepper: Insights from Multiple GWAS Models
Source: Int J Mol Sci. 2024 Nov 4;25(21):11836. doi: 10.3390/ijms252111836 (PMC11546569; doi:10.3390/ijms252111836)
Supplement: Supplementary file 1 [file ijms-25-11836-s001.zip › Supplementary Figures.pdf]

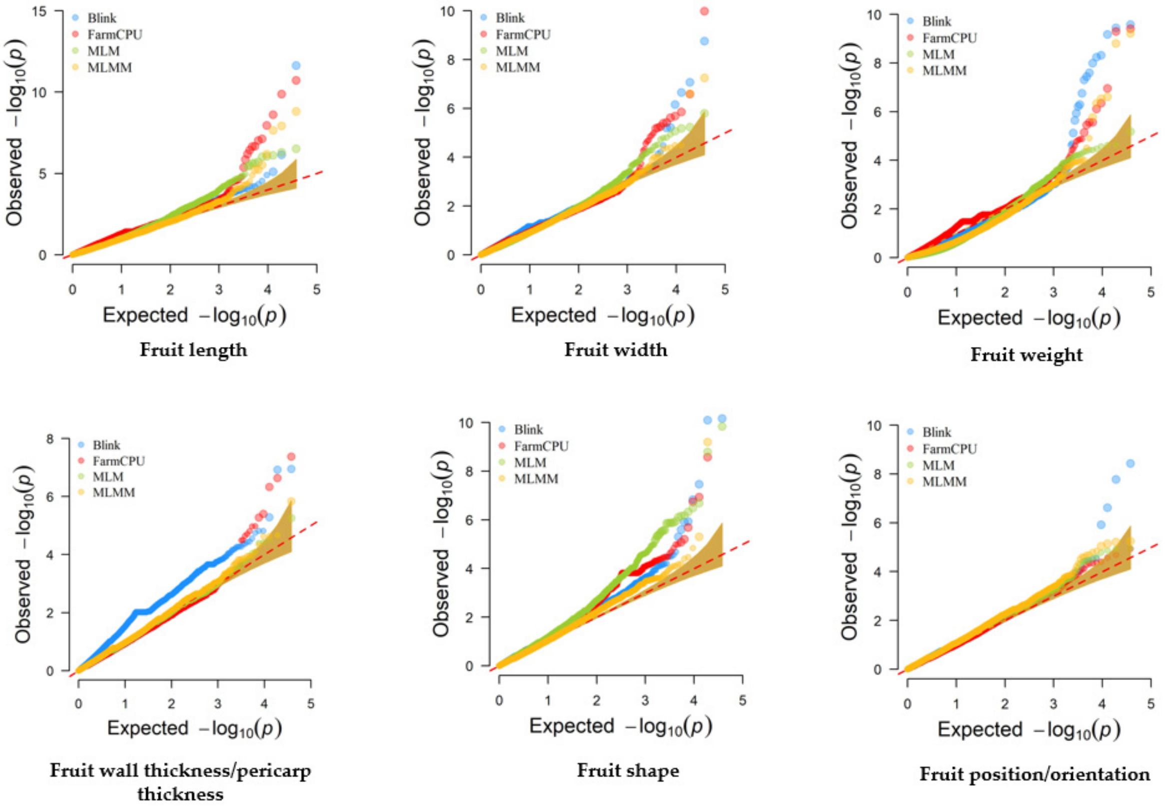

Figure S1: Quantile-Quantile (Q-Q) plots for fruit-related traits of *Capsicum* using multiple GWAS models

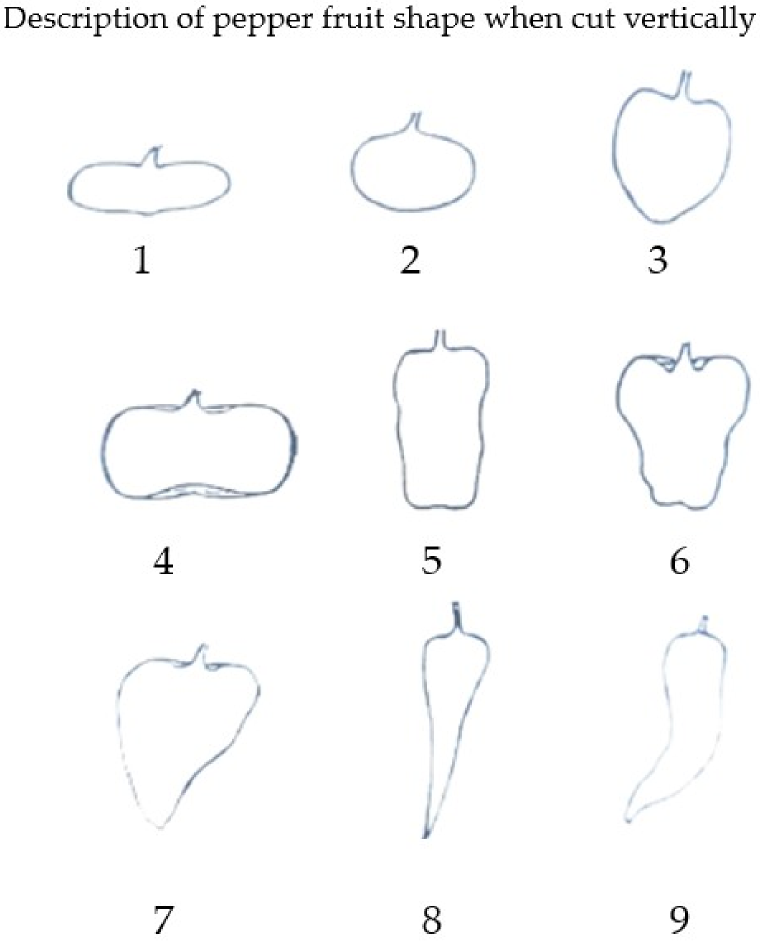

Figure S2: The fruit shape description of *Capsicum* (1 = oval (horizontally laying), 2 = round, 3 = heart-shaped, 4 = square, 5 = rectangular, 6 = trapezoid, 7 = triangle, 8 = narrow triangle, 9 = trumpet).
